# Thermodynamics of Protonation and Prototropic Equilibria in Simple *para*- and Di-*meta*-Substituted Phenols

**DOI:** 10.1021/acsomega.5c06893

**Published:** 2025-09-15

**Authors:** Andrea Kováčová, Martin Michalík, Horst Hartmann, Vladimír Lukeš

**Affiliations:** † Faculty of Chemical and Food Technology, Slovak University of Technology in Bratislava, Radlinského 9, SK-812 37 Bratislava, Slovakia; ‡ Fakultät für Chemie und Lebensmittelchemie, 216561 Technische Universität Dresden, D-010 62 Dresden, Germany

## Abstract

The systematic *ab initio* G4 study of *para*- and di-*meta*-substituted phenol derivatives is presented for the gas phase and the model water environment. The thermodynamics of proton addition was considered based on calculated proton affinities for unprotonated and monoprotonated compounds. The effect of substitution on the enthalpy balance shows a linear dependence with Hammett constants for O-protonation of the neutral compounds where the aromatic character of the molecules is preserved. Depending on the substitution and the extent of protonation, O-protonation proves to be thermodynamically less favorable than C-protonation in the monoprotonated species. It was demonstrated that the highest exothermic character of protonation was found for the gas-phase and neutral keto tautomers forming under flash photolysis that have the best ability to accept a proton. The theoretical reaction Gibbs free energies were used for the quantification of prototropic tautomerism between C-protonated species and enol–keto equilibria occurring in the suggested reaction scheme. The obtained results indicate a significant change in the tautomeric equilibria between the neutral and protonated compounds. Presented theoretical calculations could be helpful in the identification of intermediates occurring in various chemical reactions of phenols in strong acidic environments.

## Graphical abstract

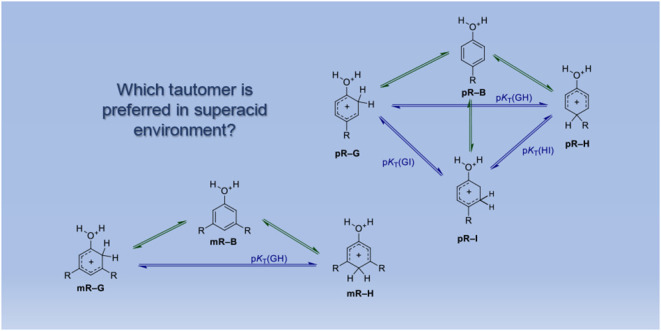

## 1 Introduction

Prototropic tautomerism, as the interconversion of constitutional isomers via proton transfer,^1^ is one of the most puzzling and interesting phenomena in chemistry. Tautomers resemble a chameleon in their behavior, as they can change from a stable structure to a different one by a change of environment. Once the original conditions are restored, they revert again. The tautomerism of phenols exhibits a specific position among enol–keto equilibria occurring in organic compounds. In contrast to cyclic and acyclic aliphatic derivatives, where the keto forms are favored,^2–4^ in the series of phenols, the enol forms are preferred under standard situations.^5^ The key effect which is responsible for the stabilization of the enol tautomer is aromaticity. When considering simple 2,4-cyclohexadienone or 2,5-cyclohexadienone,^6^ the corresponding enol tautomer is the thermodynamically more stable molecule with effective π-electron delocalization. The stabilization gained by forming an aromatic ring is sufficient to make phenol the exclusive tautomer present in the equilibrium. The equilibrium between tautomers is a dynamic process accompanied by very fast proton transfer which can be observed experimentally using nuclear magnetic resonance (NMR) techniques. The appearance of phenol–dienone tautomerism can be modulated by anellation with other aromatic rings or substitution.^7^ The presence of substituents exhibiting a marked negative conjugation effect can lead to a decrease in the energy of aromatic delocalization. This may cause a redistribution of the electron density in the molecule, with the formation of a quinonoid bond system. An electronegative group takes part in the transfer of a proton from the phenolic hydroxyl group. The keto tautomers of phenols are posed as reactive intermediates in various reactions, e.g., nucleophilic additions,^8^ oxidative NIH-shift metabolism, Kolbe–Schmitt and Reimer–Tiemann reactions,^9^ and Photo–Fries rearrangement.^10,11^

Phenol derivatives in acidic media may undergo protonation. The proton addition process can, in principle, lead to the formation of several alternative intermediates between which prototropic tautomerism can occur. For example, the protonation of phenol may occur either at the oxygen atom to form an oxonium ion or at the phenyl ring to form a protonated carbonyl moiety.^12^ The corresponding gas-phase experiments based on the mass spectroscopic methods or the photodissociation infrared spectra reveal that under the experimental conditions, the protonation occurs at the hydroxyl group and at the ring in *para* or *ortho* positions. This indicates that the addition of a small proton to phenol may be a nonselective process which obeys statistical rules. The ^1^H and ^13^C NMR spectroscopic data performed in acidic media at very low temperatures clearly demonstrated that the reaction of investigated phenol derivatives with strong Brönsted acids^13,14^ can result in O- and C-protonated isomers. The low temperatures ensure that proton exchange between the relevant phenol atoms is slowed down. The O-protonated oxonium cation is a charge-delocalized ion. The C-protonated phenol involves π-electrons and leads to a charge-delocalized cyclohexa-2,5-dien-1-ylideneoxonium ion.^15^ The experiments indicated that O-protonation is favored in weaker acidic media, while C-protonation was usually achieved in stronger superacidic environments. The presence of prototropic tautomerism between C-protonated species has also been evidenced by NMR experiments.^13^ This opened a new view of the reactivity of phenol derivatives. Despite these findings, the thermodynamics of protonation and internal proton transfer has not yet been systematically investigated. To the best of our knowledge, the energetics of the formation of the oxonium structure of phenol and its monosubstituted derivatives have received particular attention in several theoretical and experimental works.^16,17^ Studies have considered only the monoprotonated forms of these molecules. In view of this fact, we decided to investigate theoretically the processes mentioned above in parental phenol (Figure S1) and its simple *para*-substituted and di-*meta*-substituted derivatives (Figures 1 and 2). The substituents were chosen so that protonation occurs exclusively on the OH group or aromatic ring.

**Figure 1:**
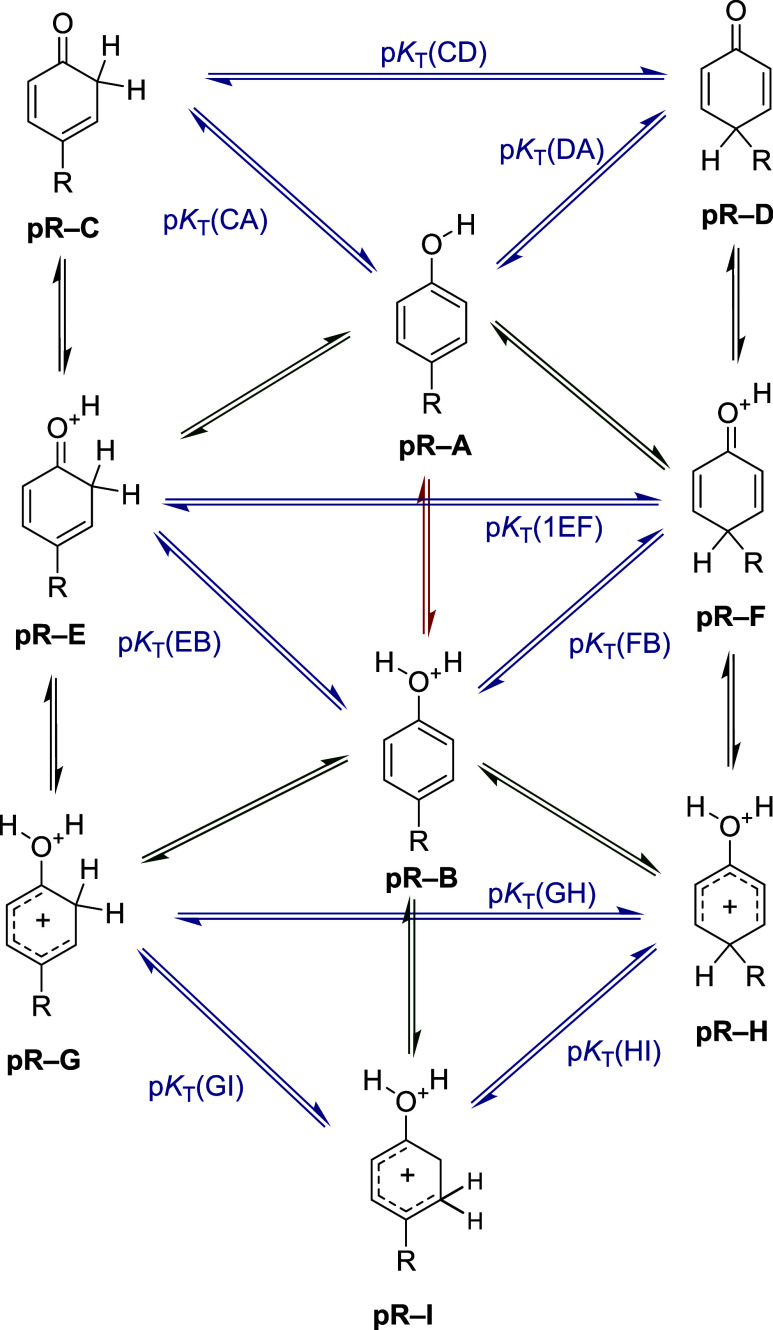
Studied protonation reactions and prototropic equilibria occurring in *para*-substituted phenol derivatives (R = CN, CF_3_, F, Cl, Br, and CH_3_).

**Figure 2:**
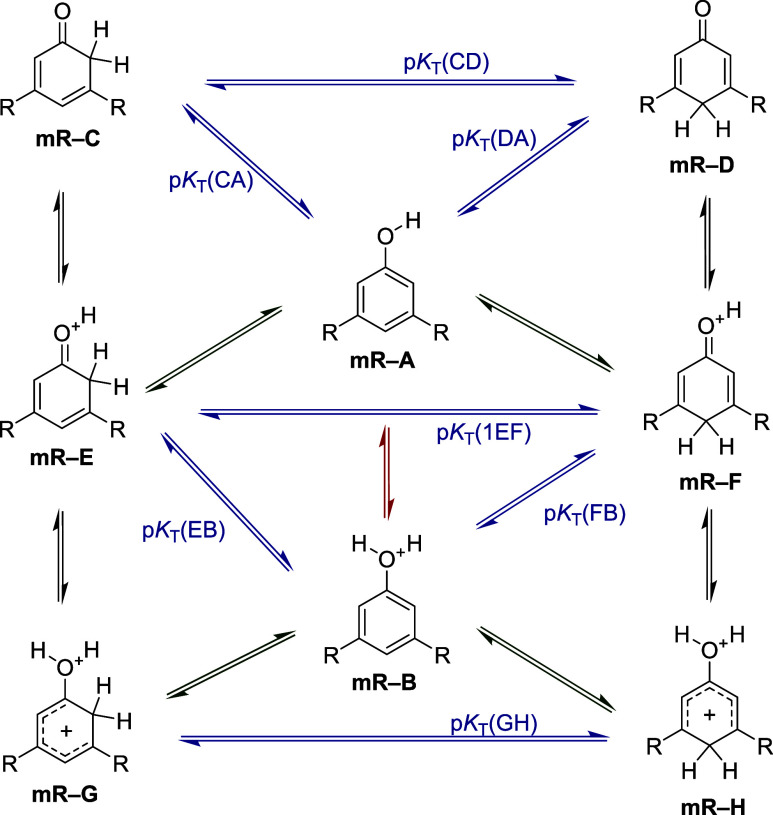
Studied protonation reactions and prototropic equilibria occurring in neutral and protonated di-*meta*-substituted phenol derivatives (R = CN, CF_3_, F, Cl, Br, and CH_3_).

This work will be primary focused on the formation of thermodynamically preferred mono- and double-protonated phenol-type species, which can occur in strongly acidic media. The theoretical gas-phase proton affinities obtained in this work will be contrasted with published available experimental and theoretical results. The thermodynamics of possible prototropic equilibria in the suggested reaction scheme will be discussed based on the reaction Gibbs free energies. The theoretical enol–keto tautomerization constants (p*K*_T_) occurring between the nonprotonated species will be evaluated for the model water environment. Finally, possible consequences on the reaction mechanisms of the studied phenol derivatives in acidic environments will be outlined in the perspective of the site of protonation and possible prototropic tautomerism.

## 2 Calculation Details

Theoretical calculations were performed using Gaussian 09 software^18^ by the composite *ab initio* Gaussian-4 level (G4) of theory.^19^ In the case of G4 theory, the optimal geometries are calculated using the density functional theory based on the 3-parametric hybrid B3LYP functional^20^ and 6–31G­(2df,p) basis set.^21^

The solvent effect contribution (water) was described using the implicit solvent model SMD (Solvation Model based on the quantum mechanical charge Density of a solute molecule interacting with a continuum).^22^ This model is based on the generalized Born equation, which is represented by an approximation of the Poisson equation suitable for arbitrary cavity shapes. The Gibbs free energies were evaluated for a temperature of 298.15 K and a pressure of 101.325 kPa. It was shown by McCann et al. that G4 provided the most accurate Gibbs free energies of tautomerization of various organic compounds.^23^ In general, the G4 method provides better agreement with experimental thermodynamic quantities (PA, IP) for organic molecules than lower versions of G*n* methods and DFT methods alone.^19^ Nevertheless, given the scaling of the G4 method in our work, the selected approach aimed at pure thermodynamics did not include bulky counterions and explicit solvent molecules. For the sake of comparison, density functional theory (DFT) employing the M06–2X^24^ functional was used in combination with the 6–311++G­(d,p)^25^ basis set. The vibrational analysis included in the calculation process showed no imaginary frequencies, confirming the geometry corresponding to the lowest energy minimum. The optimal geometries were visualized using the Jmol viewer.^26^

## 3 Results and Discussion

The investigated molecules with a higher number of atomic substituents exhibited a greater variety of conformers, which differ in the mutual spatial orientation of these groups. This conformational variability was particularly notable for molecules containing methoxy and hydroxyl groups or for those with protonated carbon atoms at the *ortho* or *meta* positions of an aromatic ring. For further study, the conformations with the lowest Gibbs energy value were used (see the Supporting Information). All optimal gas-phase geometries of conformations under study for phenol, ***m*** **Me-X**, and ***p*** **Me-X** are depicted in Figure S2. In the case of C-protonated isomers, both hydrogen atoms are over the plane of the benzene ring. The general geometries for F, Cl, Br, and CN derivatives are analogical with parent phenol species and those for CF_3_ derivatives are consistent with ***m*** **Me-X** and ***p*** **Me-X**. For cyano derivatives, the C (aromatic ring)–CN atoms are linearly arranged. Analogous geometries of the molecules were also obtained from calculations in the implicit solvent model.

### 3.1 Protonation Reactions

Within our study, ten protonation reactions were considered according to Figure 1 and nine reactions are depicted in Figure 2 where the proton is added to hydroxyl (−OH) and keto (C=O) groups or the CH part on the aromatic ring. All of these processes can be expressed by one general reaction

1 $$X+H^{+}\to {\mathrm{XH}}^{+}$$

The corresponding proton affinities (PAs) are calculated from the difference of the standard enthalpy of the original molecule (X) and the proton (H^+^) and the standard enthalpy of the protonated molecule (XH^+^)­

2 $$\mathrm{PA}=H\left(X\right)+H\left(H^{+}\right)-H\left({\mathrm{XH}}^{+}\right)$$

The gas-phase value for *H*(H^+^) is −6.2 kJ mol^–1^ ^27^ and for the water environment, we used the value of −1056 kJ mol^–1^. This value was estimated from the G4­(SMD) calculation, and it represents the enthalpy of the reaction H_2_O­(l) + H^+^(g) → H_3_O^+^(l) in water.^28,29^ The investigation commenced with the confirmation that protonation of the hydroxyl group of all parental neutral molecules (***m*** **R-A** and ***p*** **R-A**) in the gas phase is an exothermic process. A general overview of the results is compiled in Table 1. For example, the PA value of unsubstituted phenol (see the reaction **H-A** + **H** ^**+**^ → **H-B**) is 746 kJ mol^–1^ and it is even more exothermic when a proton is instead added to the carbon atom of phenol in the *ortho* position (**H-A** + **H** ^**+**^ → **H-E**) or in the *para* position (**H-A** + **H** ^**+**^ → **H-F**). The corresponding PA values for phenol in the gas phase are 798 kJ mol^–1^ for the product **H-E** and 812 kJ mol^–1^ for the product **H-F**. These results are in perfect agreement with the less accurate G3­(MP2) value (811 kJ mol^–1^) of Beelen et al.^16^ The proton affinity of a gaseous molecule is commonly obtained by studying ion–molecule equilibria either under the low-pressure conditions characteristic of FT-ICR instruments or with the use of high-pressure mass spectrometry.^30^ Experimental values reported in the literature for phenol were in the range of 816 to 818 kJ mol^–1^.^31–33^ In addition, previous research by Tishenko et al. has provided theoretical support for the prevalence of “*para*-protonation” and the subsequent formation of **H-E** products.^34^ The calculated PA value at the B3LYP/6–31+G­(d,p) level of theory is 824 kJ mol^–1^ and *ab initio* CCSD­(T)/6–311++G­(d,p) gives the value of 819 kJ mol^–1^. Furthermore, a close examination of the chemical structure of the O-protonated phenol cation (**H-B**) reveals a capacity to capture the next proton on the carbon atoms of an aromatic ring. For subsequent **H-F** and **H-E** cations, protonation of the =OH^+^ group is supposed. The exothermic character of these reactions is however less than half of the former. The predicted gas-phase PAs corresponding to the formation of the species **H-G** for both locations on the aromatic ring are 297 kJ mol^–1^, and those for species **H-H** and **H-I** are 308 and 307 kJ mol^–1^, respectively. Nevertheless, the other routes to products **H-G** and **H-H** from the protonated forms of **H-E** and **H-F** are associated with slightly lower proton affinities of 246 and 242 kJ mol^–1^, respectively. Interestingly, the O-protonation in keto tautomers **H-C** and **H-D** is energetically preferable. The PAs are 874 kJ mol^–1^ for **H-C** + **H** ^**+**^ → **H-E** and 882 kJ mol^–1^ for **H-D** + **H** ^**+**^ → **H-F**. A systematic evaluation revealed significant variations in the enthalpic equilibrium of protonation reactions within a water environment that was selected for the study. Specifically, the exothermic character of the studied reactions shifted to an endothermic one, e.g., the PA for the formation of the cation **H-F** from **H-A** is negative, i.e., −24 kJ mol^–1^. A slightly lower value of −35 kJ mol^–1^ exhibits the formation of **H-E** and **H-B** products. In addition, the reactions in water favor C-protonation over O-protonation (see Table S2). When implicit solvent interactions are included, the total enthalpies of individual species are reduced. Analysis of the calculated molecular enthalpies indicates that the total solvation contribution decreases the total molecular enthalpy. The minimal solvation contribution between the nonprotonated and protonated forms is −223 kJ mol^–1^ (**H-D** vs **H-F**) and the maximal contribution is −702 kJ mol^–1^ (**H-E** vs **H-G**). The solvation effect evidently plays a major role in protonated molecules. Nevertheless, a key influence on the thermodynamics is the solvation enthalpy of the proton in the solvent. The transfer of a proton from the hydrated state to the phenol molecule’s corresponding form is a fundamental step in this process. The theoretical calculation of the solvation enthalpy in strong acids encounters the natural limits of the implicit model since no amenable parameters are available for these conditions. For anhydrous strong acids, an increased concentration of H^+^ ions can be expected to stimulate the formation of diprotonated cationic forms of phenol. In addition, the proton in a strong acidic environment tends to be weakly solvated. For example, the estimated solvation energy of the proton in liquid hydrogen fluoride is in the interval −941 to −983 kJ mol^–1^.^35^ This value is considerably lower than the proton hydration energy in water. In accordance with the above discussion, it is plausible to suppose that the higher chemical activity of protons in water-free super acids will decrease the endothermic character of individual studied reaction steps.

**Table 1:** Calculated G4 Proton Affinities of Phenol (H) and Relative Proton Affinities of Derivatives with Respect to Phenol in the Gas Phase in kJ mol^–1^

**R**	**A → B**	**A → E**	**A → F**	**B → G**	**B → H**	**B → I**	**C → E**	**D → F**	**E → G**	**F → H**
**H**	746	798	812	297	308	307	874	882	246	242
***p*** **Me**	11	13	–1	24	4	49	1	4	20	16
***p*** **F**	–13	–25	–61	–29	–77	10	–26	–47	–18	–29
***p*** **Cl**	–14	–23	–47	–11	–56	31	–22	–36	–4	–24
***p*** **Br**	–18	–22	–32	–1	–14	42	–20	–28	1	0
***p*** **CF**_**3**_	–36	–40	–47	–28	–35	–28	–35	–39	–25	–24
***p*** **CN**	–46	–57	–84	–46	–79	–31	–49	–54	–36	–41
***m*** **Me**	17	51	47	93	80		35	31	58	50
***m*** **Br**	–32	–4	–10	71	52		–11	–19	43	30
***m*** **Cl**	–34	–5	–11	58	39		–13	–21	28	17
***m*** **F**	–41	–9	–17	21	2		–19	–30	–11	–22
***m*** **CF**_**3**_	–58	–72	–71	–52	–52		–71	–70	–39	–38
***m*** **CN**	–83	–97	–98	–54	–62		–89	–90	–41	–47

The introduction of a substituent modifies the electronic structure of the ring system, thereby governing the thermodynamics of protonation, especially upon the loss of aromaticity. In this context, derivatives considered in this study with both *meta* positions substituted showed a significantly wider spread of proton affinity values compared to that of *para* isomers. In the case of O-protonation of the parent molecule (see the reaction **A** + **H** ^**+**^ → **B**), the role of the substituent can be analyzed using Hammet constants^36^ because both the reactant and the product are aromatic molecules (see Table S2).

Figure 3 presents the correlation between Hammett constants (σ_m_ and σ_p_, shortly denoted as σ_m,p_) and PAs calculated for the gas phase. For this type of analysis, we have used theoretically evaluated Hammett substituent constants.^37^ The equations obtained from the linear regression are as follows

3 $$PA/{\mathrm{kJ mol}}^{-1}=-22.1\times \sigma_{p}+747\left(\mathrm{gas}\right)$$

4 $$\mathrm{PA}/{\mathrm{kJ mol}}^{-1}=-22.8\times \sigma_{m}+763\left(\mathrm{gas}\right)$$

**Figure 3:**
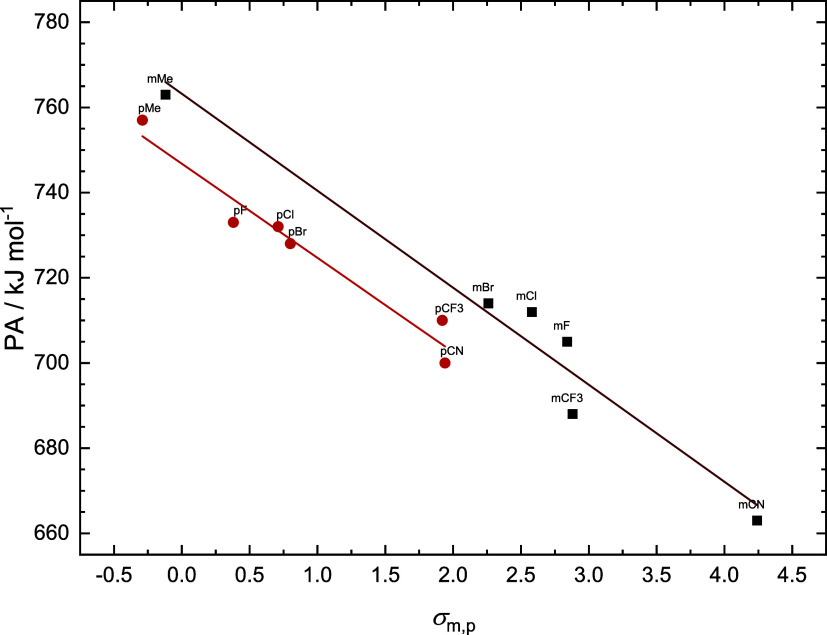
Correlation between proton affinities (PAs) of the protonation reaction **R-A + H** ^**+**^ **→ R-B** on the Hammett constants calculated for the gas phase.

Coefficients of determination for gas-phase/water data reached 0.95 and 0.96 for *para* and *meta* substituents, respectively. For di-*meta*-substituted derivatives, we used double the value of the Hammett constant for each substituent in the linear dependence. The approximately identical magnitude of the slopes suggests that one substituent in the *para* position will affect the PA value as much as two substituents in the *meta* position. The influence of the environment on the substitution effect can be estimated from the comparison of slopes and intercepts.

5 $$\mathrm{PA}/{\mathrm{kJ mol}}^{-1}=-5.9\times \sigma_{p}-35\left(\mathrm{water}\right)$$

6 $$\mathrm{PA}/{\mathrm{kJ mol}}^{-1}=-6.3\times \sigma_{m}-35\left(\mathrm{water}\right)$$

Coefficients of determination are 0.86 and 0.93 for the *para* and *meta* substituents, respectively. The slope values calculated for water indicate that the environment significantly modulates the effect of the substituent on PAs. On the other hand, the qualitative conclusion resulting from the comparison of the effect of *meta* and *para* substitution is the same as in the gas phase.

Gas-phase calculations provide fundamental insights into intrinsic molecular acidity, establishing useful baselines for comparative studies without environmental effects. The solvent effect resulted in a change in the slope of the trend lines compared with the gas phase. Furthermore, it was determined that superior results were obtained from the gas phase for *para* derivatives and from the water environment for *meta* derivatives, respectively. Although the effect of the substituent tends to be best described for the formation of the oxonium cation **B**, this reaction pathway, which preserves the aromatic character of the product, is thermodynamically less preferred. Notwithstanding, experimental evidence has been found to support the existence of an oxonium cation in a gas phase under certain conditions.

Next, as the reaction schemes proceed via nonaromatic intermediates, the direct resonance interaction between the substituent and the reaction center invalidates the assumptions of the standard Hammett equation. For this reason, it is reasonable to compare ΔPA values representing the difference between the PA of the substituted phenol and the phenol itself. The data contained in Table 1 reveal that the formation of the **E** product from parental **A** relates to smaller proton affinities for electron-withdrawing substituents. The cyano derivative exhibits a minimal ΔPA value of −97 kJ mol^–1^. A similar situation was found for the formation of the **F** products. Interestingly, even in the case of ***p*** **R-A** derivatives, where the steric shielding is in the *para* position, the C-protonation is exothermic in nature. The methoxy group in *meta* derivatives is usually associated with the highest PAs. Analogous to the parent phenol (**H-A**), the formation of diprotonated species is characterized by proton affinities that are approximately 2-fold lower. For instance, conversion of the isomer **B** to **G** exhibits the lowest PA value. In contrast, for *para*-substituted derivatives, the formation of products **H** and **I** is the most enthalpically favorable pathway, with the specific preference between them being determined by the substituent. Likewise, the presence of a water solvent leads to a significant change in the enthalpy balance of the studied reaction. Interestingly, the exothermic nature of the reaction in water remains for the **C** + **H** ^**+**^ → **E** (except for ***m*** **CN**) and **D** + **H** ^**+**^ → **F** reactions. Furthermore, a clear and consistent trend in the proton affinity (PA) values is evident across the halogen series (F, Cl, Br) derivatives.

According to Table 1, the PA values for almost all reactions involving ***m*** **Cl** are considerably lower compared to that of ***m*** **Me**. It makes sense that ***m*** **Cl** would not be as willing to exchange hydrogen protons with acids. This finding is supported by the experimental results presented by Hartmann^13^ where only 42–46% deuteration occurred for ***m*** **Cl** but it was 92% for ***m*** **Me**. The results listed in Table 1 also show that for ***m*** **Me**, we obtained slightly lower PA values for **B** + **H** ^**+**^ → **H** and **B** + **H** ^**+**^ → **G**, which are relevant for a very acidic environment. This finding provides an explanation for the lower degree of deuteration observed. Given the chemical nature of the CN group, its protonation can also be hypothetically assumed, which would open up further branching possibilities for reaction mechanisms. To the best of our knowledge, we have found an experimental study that showed only the protonation of the OH group and the transfer of a proton to the benzene ring in ***p*** **CN**.^38^ The CN group did not directly participate in this process.

Although the G4 method is considered the gold standard for calculating thermodynamic quantities, it is useful to perform a comparative calculation using the DFT approach. The results of M06–2*X*/6–311++G­(d,p) proton affinities can be found in Table S3. As shown in Figure S4, the comparison of G4 and DFT shows linear trends with a coefficient of determination better than 0.999. The slopes are close to one, and there are differences in intercepts. A slightly greater dispersion of points is evident in the results obtained using the implicit solvent model. This may be due to insufficient coverage of the solvation contributions of the implicit model for charged species.

### 3.2 Prototropic Equilibrium

Isomerization of the parent phenol (**H-A**) to its keto tautomers (**H-C** and **H-D**) requires the disruption of aromaticity and is therefore strongly endergonic (Δ*G* > 0). The reaction Gibbs free energies are calculated as the difference between the Gibbs free energies of enol and keto forms

7 $$\Delta_{r}G=G\left(\mathrm{keto}\right)-G\left(\mathrm{enol}\right)$$

The corresponding results for the gas phase are 72 kJ mol^–1^ for Δ_r_ *G*_T_(**AC**) and 69 kJ mol^–1^ for Δ_r_ *G*_T_(**AD**) (see Table 2). Implicit aqueous solvation preferentially stabilizes the more polar keto tautomers, thereby slightly reducing the free energies of tautomerization to 72 kJ mol^–1^ Δ_r_ *G*_T_(**AC**) and 65 kJ mol^–1^ Δ_r_ *G*_T_(**AD**). The results for water are comparable with the recently published theoretical calculations at the density level of theory M06–2X­(SMD = water)/6–311++G­(d, p).^7^ The small positive value of Δ_r_ *G*_T_(**DC**) = 4 kJ mol^–1^ in the gas phase or 7 kJ mol^–1^ in water indicates the preference of the **H-D** tautomer. The appropriate hydroxy forms ***p*** **R-A** are calculated in accordance with the experiments to be significantly more stable than their corresponding keto forms ***p*** **R-C** and ***p*** **R-D**. Nevertheless, these keto tautomers have been identified experimentally by NMR spectroscopy.^39^

**Table 2:** Calculated Reaction G4 Gibbs Free Energies (Δ_r_ *G*) of the Studied Tautomeric Reactions in kJ mol^–1^

	Δ_r_ *G*(**AC**)		Δ_r_ *G*(**AD**)		Δ_r_ *G*(**BE**)		Δ_r_ *G*(**BF**)		Δ_r_ *G*(**DC**)		Δ_r_ *G*(**EF**)		Δ_r_ *G*(**GH**)		Δ_r_ *G*(**GI**)		Δ_r_ *G*(**HI**)	
R	gas	water	gas	water	gas	water	gas	water	gas	water	gas	water	gas	water	gas	water	gas	water
**H**	72	72	69	65	–50	0	–63	–10	4	7	–13	–11	–10	–8	–9	5	1	13
***p*** **Me**	68	70	82	73	–48	–2	–46	1	–14	–3	2	3	11	11	–36	–12	–48	–23
***p*** **F**	71	73	82	81	–38	8	–16	29	–10	–9	22	21	39	37	–48	–12	–87	–49
***p*** **Cl**	73	74	78	77	–43	10	–33	21	–5	–3	10	11	32	25	–51	–6	–83	–32
***p*** **Br**	74	75	72	72	–51	11	–55	14	2	3	–4	4	1	16	–53	–1	–53	–17
***p*** **CF**_**3**_	70	76	71	77	–46	6	–50	5	–1	–1	–5	–2	1	8	–7	18	–8	10
***p*** **CN**	79	79	98	96	–40	11	–27	29	–18	–17	13	17	22	23	–24	11	–47	–12
***m*** **Me**	67	56	62	48	–76	–27	–86	–39	4	8	–10	–12	2	–3				
***m*** **Br**	66	69	59	61	–77	4	–85	–3	7	7	–8	–7	8	0				
***m*** **Cl**	65	67	58	58	–78	–1	–86	–11	7	9	–8	–10	7	–2				
***m*** **F**	62	62	55	52	–81	–17	–87	–24	8	10	–6	–7	8	4				
***m*** **CF**_**3**_	76	79	73	73	–34	27	–47	13	4	6	–13	–14	–9	–8				
***m*** **CN**	80	82	77	78	–36	31	–49	22	4	4	–13	–8	–3	–10				

The expensive G4 approach is currently considered a method designed to calculate precise Gibbs energies. Still, the estimated deviation of reaction Gibbs energies with respect to the experiment can be as high as 10 kJ mol^–1^. In the case of the tautomeric equilibrium studied, four experimental p*K*_T_ constants for phenol and 1-naphthol (see Table S1)^40^ are available in the literature for room temperature in acidic water solutions. The correlation of these experimental values with the corresponding theoretical reaction Gibbs free energies (in kJ mol^–1^) exhibits linear dependence

8 $$pK_{T‐\exp}=1.09-0.158\times \Delta_{r}G$$

with the coefficient of determination *R* ^2^ = 0.967 (Figure S6). The nonzero intercept presumably consists of constant contributions of solvation which are not implicitly covered by the implicit SMD model. The slope and the intercept also include the constant part (1/*RT*)/ln 10, i.e., 0.175 mol kJ^–1^, at room temperature. The ratio between the slope and this constant part is 0.90. Quantitatively, this finding suggests that the free enthalpy of the reaction (Δ_r_ *G*) has been underestimated. This discrepancy is likely due to the dissolution corrections, which depend on the specific chemical structures of the keto and enol tautomers. Using the parameters from eq 8, we calculated the equilibrium constants (as scaled p*K̃*_T_ values) for the tautomers of molecules **R-A**, **R-C**, and **R-D**. These results are plotted in Figure 4 to visually assess how the molecular structure affects the tautomeric equilibrium.

**Figure 4:**
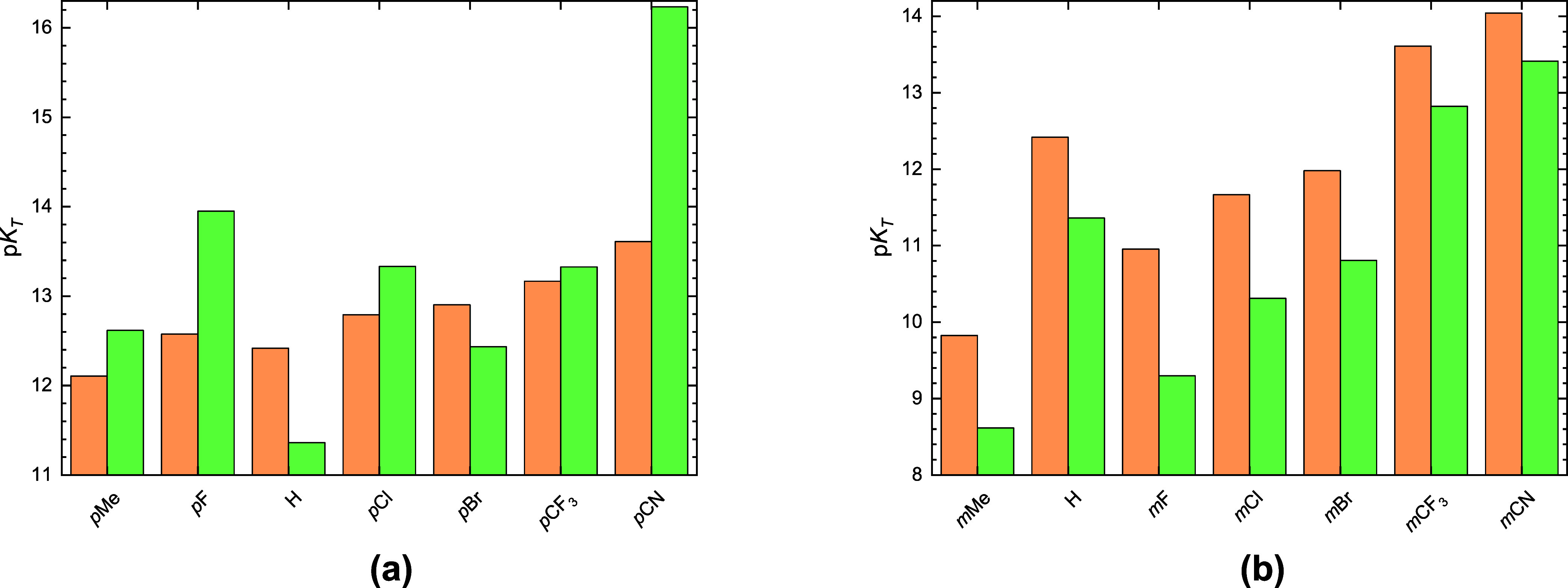
Values of scaled p*K*_T_ values of studied enol–keto tautomerization equilibria for *para*- (a) and *meta*- (b) substituted species. Notation for depicted p*K*_T_ values: green for p*K*_T_(**AC**) and orange for p*K*_T_(**AD**).

The compounds under examination are characterized by comparatively large tautomeric constants, confirming that their respective keto tautomers occur at relatively low concentrations under normal circumstances. Furthermore, the results of the analysis indicate that the monosubstitution in the *para* position increases p*K*_T_ values compared to the parental phenol. The largest tautomeric constants for both equilibria exhibit ***p*** **CN**. An interesting feature of *meta* disubstitution is that it often induces contrasting effects on the p*K*_T_ values. The lowest values of 9.9 for p*K*_T_(**AC**) and 6.5 for p*K*_T_(**AD**) have methyl groups. The maximal values of 14.0 for p*K*_T_(**AC**) and 13.2 for p*K*_T_(**AD**) have cyano groups. Although keto tautomers are present in negligible amounts, the equilibrium can shift significantly upon excitation of the sample with light.^40^ Consequently, these tautomers become thermodynamically interesting in protonation reactions (Table 1).

In the gas phase, the tautomerization of the **R-B** oxonium cation to isomers **R-E** and **R-F** is thermodynamically favorable, proceeding via an exergonic pathway. (Table 2). On the other hand, solvation in water significantly alters the thermodynamics of the system, causing the Gibbs free energies of tautomerization to become near-zero or even take positive values. This shift suggests that the equilibrium either supports a more equitable population of both tautomers or favors the initial reactant. This is especially true for the strong electron acceptors, i.e., the CF_3_ and CN groups for *para* and *meta* derivatives. The subtraction of Δ_r_ *G*(**BF**) from Δ_r_ *G*(**BE**) leads to Δ_r_ *G*(**EF**), which quantifies the protolytic equilibrium between the **R-F** and **R-E** species. In the *para* derivatives, the **F** isomer dominates, while in the *meta* derivatives, the **E** isomer prevails.

As it was mentioned previously, the next addition of a proton can lead to three (see Figure 1) or two possible (see Figure 2) isomers. An analysis of the calculated gas-phase Δ_r_ *G* values for monosubstituted *para* derivatives (see Table 2) reveals that the formation of isomers is in the order **H** → **I**, **G** → **I**, and **G** → **H**. With a relative Gibbs free energy (Δ*G*) of only 1 kJ mol^–1^, the two parent phenol isomers (**H** and **I**) coexist in significant proportions, as neither is strongly favored thermodynamically. The inclusion of an implicit solvent model reduces the exergonic character of the equilibrium reactions. For example, Δ_r_ *G*(**GI**) and Δ_r_ *G*(**HI**) calculated for *p*CF_3_ are positive values, which indicate the endergonic character of the reaction. In the case of disubstituted molecules, the values of the reaction Gibbs energies vary only in smaller ranges, i.e., 2 to −8 for gas and −4 to 10 for water. Also, the stronger electron-withdrawing groups CF_3_ and CN slightly favor the formation of the H isomer over the G isomer. Based on these results, we expect that substituents in the *meta* position affect the protolytic equilibrium between the G and H isomers to a lesser degree. The relationship between G4 Gibbs energies and auxiliary DFT calculations (Table S4) indicated linear correlations for both the gas phase and the aqueous environment (see Figure S5). Similar to proton affinities, deviations are mainly evident in the intercept. Even these comparisons of Gibbs energies did not reveal significant differences in the thermodynamics of the studied substituents by two distinct computational approaches.

### 3.3 Experimental Consequences of Prototropic Tautomerism

Although a strong acid will preferentially attack the hydroxyl group due to electrostatic interactions, theoretical calculations indicate a plausible influence of prototropic tautomerism. Deuteration experiments with trifluoric acetic acid (TFA) and trifluormethanesulfonic acid (TFSA) demonstrated internal proton transfers within the aromatic ring.^13^ Theoretical calculations indicated that the thermodynamics of these processes is also influenced by the nature of the substituent. For the one protonated species in a model water environment, the **E** tautomer is preferred for *p*Me and the **F** tautomer is preferred for phenol. The **B** tautomer with protonated oxygen is thermodynamically favored for *para*-substituted molecules with electron-withdrawing groups. The **B** tautomer dominates only for CF_3_ and CN groups for di-*meta*-substituted derivatives. Addition of the second proton leads to the formation of the **H** tautomer for phenol, *p*CF_3_, and all di-*meta*-substituted derivatives. The **I** tautomer is preferred for the remaining *para* derivatives. Experimental measurements show that the population of individual tautomers depends on the type of strong acid. For instance, in *para*-cresol protonations, it was possible to estimate the actual concentration of all of the protonated tautomers in TFA as ***p*** **Me-B**/***p*** **Me-E** /***p*** **Me-F** = 55:40:5 and in HF-SbF_5_ as ***p*** **Me-B**/***p*** **Me-E**/***p*** **Me-F** = 19.0:73.5:7.5.^14^ These results agree with our calculations in the model water environment that favor the compound ***p*** **Me-E** over ***p*** **Me-B** and ***p*** **Me-F**.^7^ The other protonated keto tautomers ***p*** **Me-E** and ***p*** **Me-F** could not be established spectroscopically, but they were identified by typical chemical reactions. There are also a few studies on the tautomerism of phenols and their protonated derivatives in the series of di-*meta*-substituted compounds. These compounds exist in neutral and weak acidic solutions, as indicated by NMR measurements, in their unprotonated form as OH tautomers ***m*** **R-A**, which is also reflected in the calculated data. With respect to the protonated compounds, it is only known that 3,5-dimethylphenol (***m*** **Me-A**) is protonated in TFS at 100% on its *para* position under the formation of the compound ***m*** **Me-F**.^14^ However, this contradicts with the calculations which determined the tautomers ***m*** **Me-E** as the most stable compounds and raises the question for the actual structure of other protonated species, such as of the dihalo-substituted phenols, which should exist, according to the calculated data, preferentially as F tautomers.

Protonation of phenol or its derivatives can be the initiation step to several types of reactions that can be used in organic synthesis. The position of the proton in the molecule and the thermodynamical stability of this cation are also critical. Therefore, the theoretical data obtained can be used to verify the reaction mechanisms in acidic environments. For example, the heating of the parent phenol (**H-A**) with 10% of TFSA in water at 130 °C leads to the formation of diphenylether. An excess of acid as well as a higher temperature and longer reaction times did not improve the reaction yield.^41^ The calculated proton affinity values in the gas phase (Table 1) for phenol show that the **H-F** tautomer is the most thermodynamically stable tautomer. Similarly, the Gibbs reaction energies for tautomeric equilibria summarized in Table 2 for water indicate the thermodynamic preference of the **H-F** tautomer. Therefore, the reaction mechanism for ether formation or even the analogous O-alkylation reactions can be explained using Scheme 1. The cationic tautomer **H-F** attacks a second phenol **H-A** with its electrophilic *C*4 position under the formation of the adduct **H-X**. This adduct is subsequently transferred under the elimination of water into diphenyl ether (**H-Y**).

**Scheme 1:**
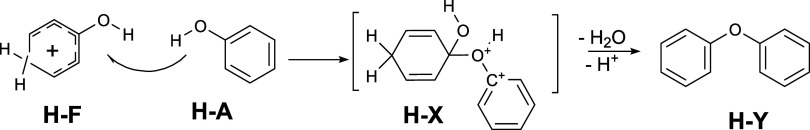
Reaction Mechanism of Phenol

Another interesting example is the bromination of *para*-cresol disolved in HSbF_6_. This reaction with bromine in a nonwater environment gives rise to the formation of 3-bromo-4-methylphenol. Theoretical calculations of Δ_r_ *G* for the gas phase reveal that the ***p*** **Me-E** and ***p*** **Me-F** tautomers would be thermodynamically relevant. According to Scheme 2, the protonated keto tautomer ***p*** **Me-E** adds a bromide ion under the formation of the adduct ***p*** **Me-X** that add, in turn, bromine under the formation of the adduct ***p*** **Me-Y** as the precursor for the generation of 3-bromo-4-methylphenol ***p*** **Me-Z**. The preferred presence of ***p*** **Me-E** in the reaction mixture is consistent with the reaction mechanism proposed in the work of Brittain et al.^42^ However, an analogous bromination process can also be assumed for the other equivalent tautomer.

**Scheme 2:**
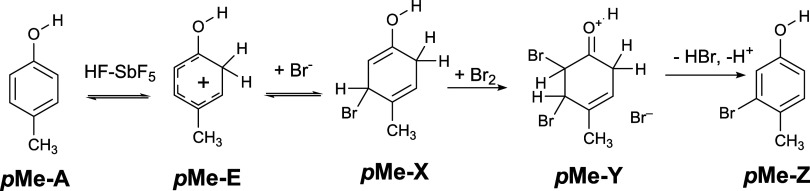
Reaction Mechanism of ***p*Me** Bromination

The chemical theory developed for the reactions of phenols in strong acids supposed the formation of double-protonated isomers, which represent key intermediates. For example, the condensation reaction of *p*-methylphenol with benzene in a superacidic (HF-SbF_5_) nonwater environment is initiated by the ***p*** **Me-I** tautomer (Scheme 3).^35^ This assumption is also in good agreement with our theoretical predictions (Table 2).

**Scheme 3:**
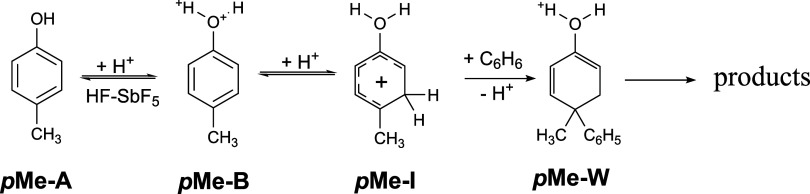
Mechanism of the Benzene Addition Reaction to ***p*Me**

## 4 Conclusions

The thermodynamics of internal proton transfer, investigated in this study at the G4 level of theory for neutral and protonated phenols and their selected *para*- and di-*meta*-substituted analogues, maps the energetic landscape of the reactions. For O-protonation, where the aromatic character of the molecules is preserved, we have demonstrated the effect of substitution using Hammett constants. Depending on the substitution and the extent of protonation, O-protonation proves to be thermodynamically less favorable than C-protonation in the relevant cases. The highest exothermic character of protonation was found for the gas-phase and neutral keto tautomers (**R-C** and **R-D**) forming under flash photolysis that have the best ability to accept a proton. The electron-withdrawing substituents decrease the proton affinities of investigated protonation reactions. The exothermic character of the studied protonation reactions is changed to endothermic within a model water environment. A key influence on thermodynamics is the solvation enthalpy of the proton in the solvent, which is required to transfer the proton from the hydrated state to the corresponding form of the phenol molecule. The theoretical reaction Gibbs free energies were used for the quantification of prototropic tautomerism between C-protonated species and enol–keto equilibria occurring in the suggested reaction scheme. The thermodynamic data presented herein should prove valuable for the identification of intermediates formed during the condensation or deuteration of phenols in strongly acidic media. Thermodynamic calculations demonstrated the feasibility of the processes under investigation under real conditions. In order to facilitate a complex investigation of the processes in question, it is advisible to undertake a thorough study of the reaction barriers involved in proton transfers. However, it is important to note that this simulation should be performed for chemical models containing specific counterions. This would enable the estimation of the kinetics of the reactions involved in tautomeric equilibria.

## Supplementary Material



## Data Availability

The data that support the findings of this study are available in the article’s Supporting Information.

## Notes

Enol and keto tautomers occurring in nonprotonated and protonated phenol (Figure S1); the studied tautomerism reaction and the available experimental p*K*_T_ values in water for naphthalen-1-ol (Scheme S1); calculated B3LYP/6–31G­(2df,p) optimal geometries (Figure S2, S3); correlation between G4 and M06–2*X*/6–311++G­(d, p) for proton affinities (Figure S4) and Gibbs energies (Figure S5); correlation between available experimental p*K*_T_ values and calculated G4 Gibbs free energies of the studied tautomerization reaction (Figure S6); calculated Gibbs free energies and experimental p*K*_T_ (Table S1); calculated G4 proton affinities (Table S2); and calculated M06–2*X*/6–311++G­(d, p) proton affinities (Table S3) and Gibbs free energies (Table S4) (PDF)
