# Supplementary material for: Thermodynamics of Protonation and Prototropic Equilibria in Simple para- and Di-meta-Substituted Phenols
Source: ACS Omega. 2025 Sep 15;10(37):43209–18. doi: 10.1021/acsomega.5c06893 (PMC12461337; doi:10.1021/acsomega.5c06893)
Supplement: Supplementary file 1 [file ao5c06893_si_001.pdf]

Thermodynamics of protonation and prototropic equilibria in simple *para*- and di-*meta*- substituted phenols

Andrea Kováčová[a)*], Martin Michalík[a)], Horst Hartmann[b)], Vladimír Lukeš[a)]

[a)] Faculty of Chemical and Food Technology, Slovak University of Technology in Bratislava, Radlinského 9, SK-812 37 Bratislava, Slovakia

[b)]Fakultät für Chemie und Lebensmittelchemie, Technische Universität Dresden, D-010 62 Dresden, Germany

* Corresponding author: andrea.kovacova@stuba.sk

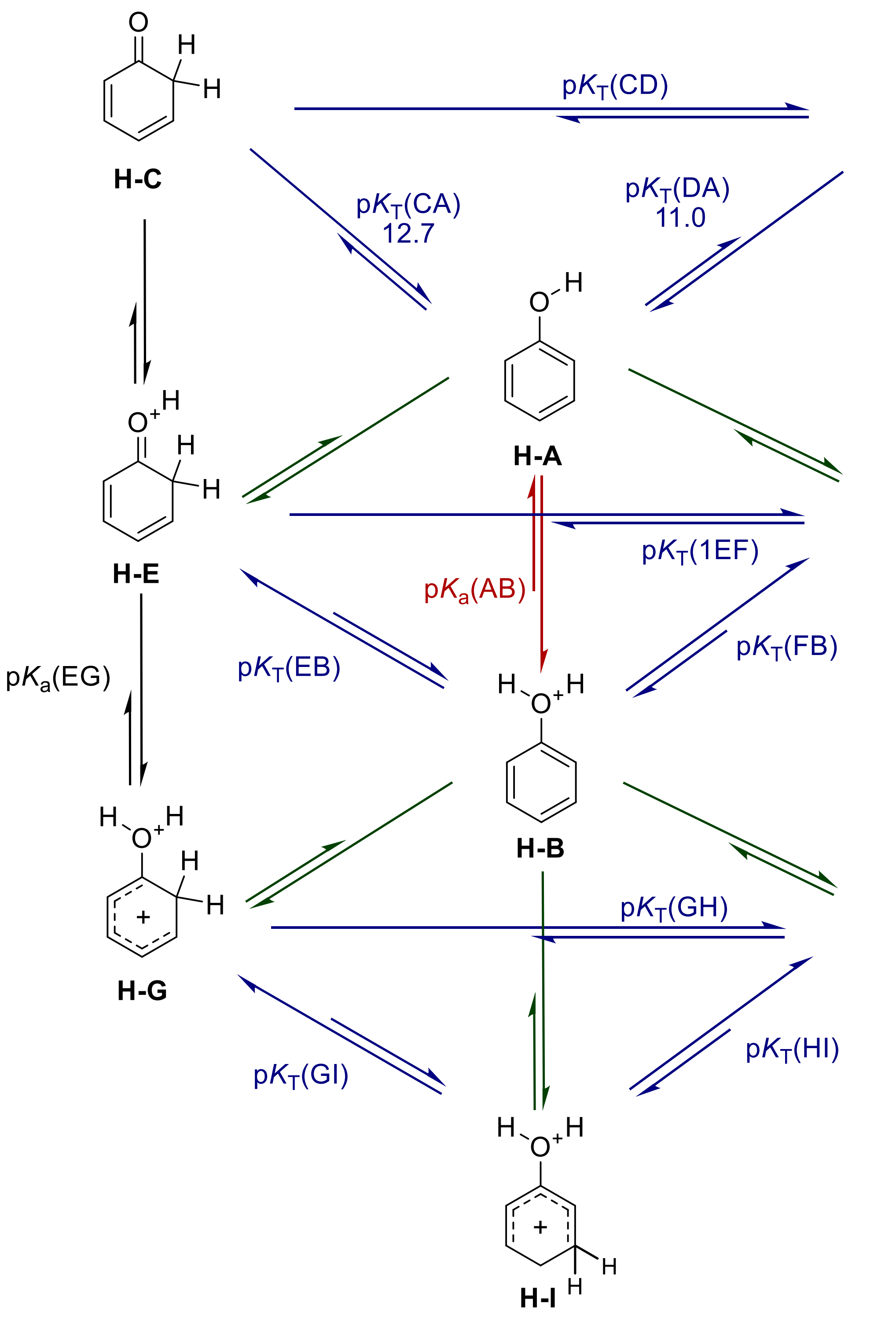

Figure S1. The enol and keto-tautomers occurring in non-protonated and protonated phenol (**H**).

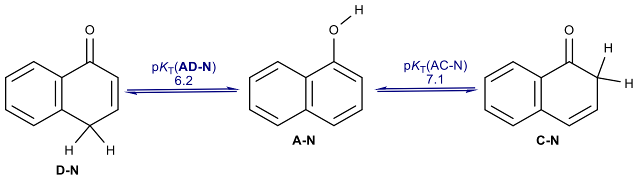

Scheme S1. The studied tautomerism reaction and the available experimental pK_T values in water for naphthalen-1-ol (N).[1]

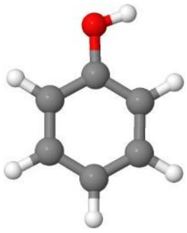

H-A

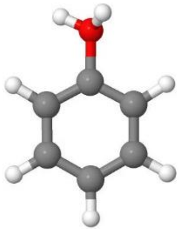

H-B

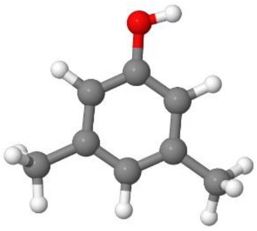

*m*Me-A

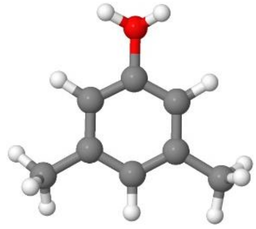

*m*Me-B

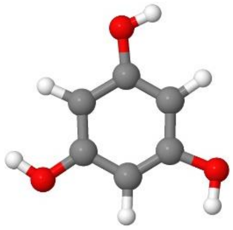

*m*OH-A

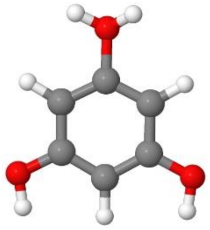

*m*OH-B

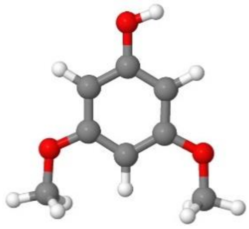

*m*OMe-A

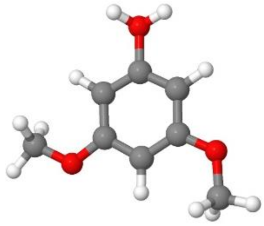

*m*OMe-B

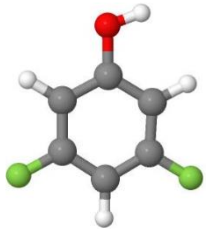

*m*F-A

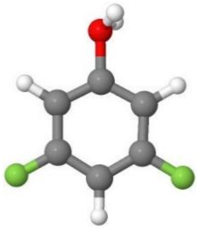

*m*F-B

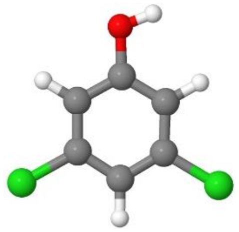

*m*Cl-A

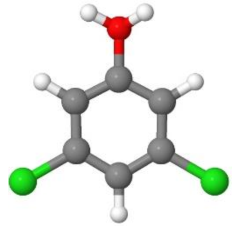

*m*Cl-B

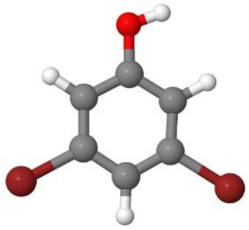

*m*Br-A

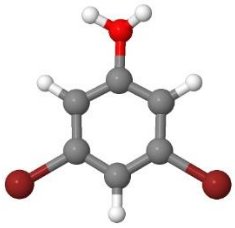

*m*Br-B

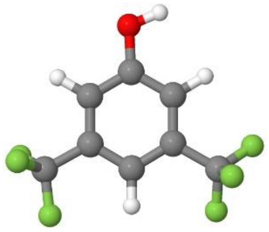

mCF_3-A

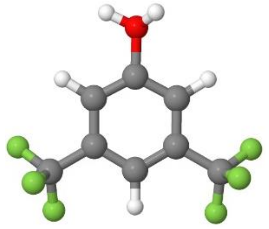

mCF_3-B

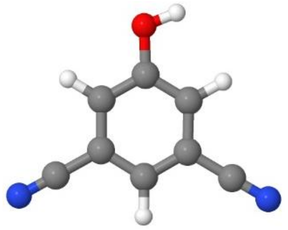

*m*CN-A

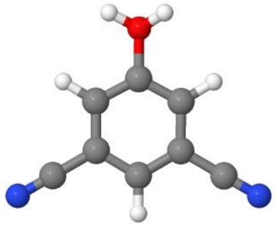

*m*CN-B

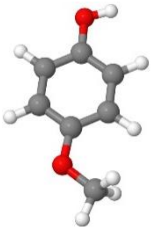

*p*OMe-A

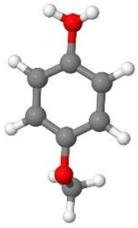

*p*OMe-B

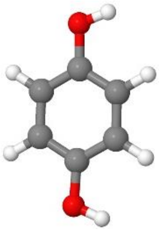

*p*OH-A

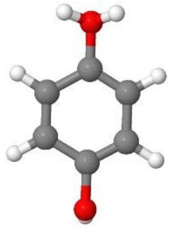

*p*OH-B

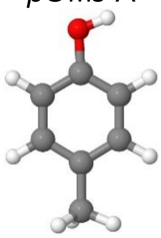

*p*Me-A

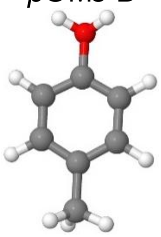

*p*Me-B

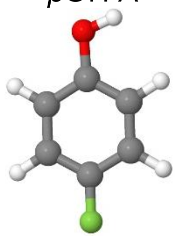

*p*F-A

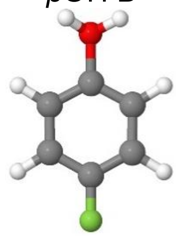

*p*F-B

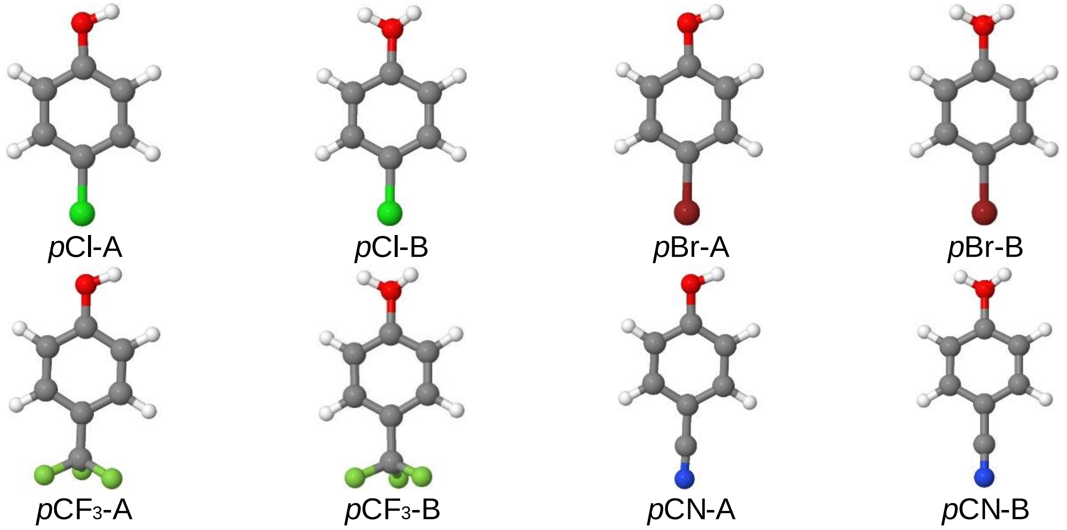

Figure S2. Calculated B3LYP/6-31G(2df,p) optimal geometries for studied species in neutral (**A**) and protonated cationic (**B**) forms.

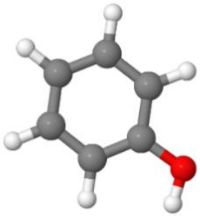

H-A

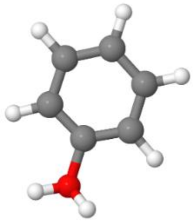

H-B

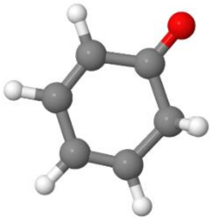

H-C

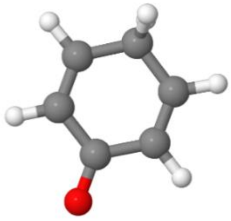

H-D

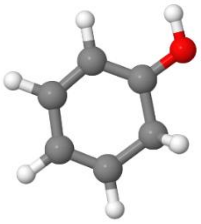

H-E

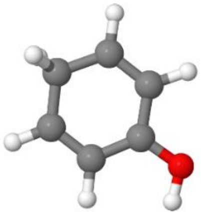

H-F

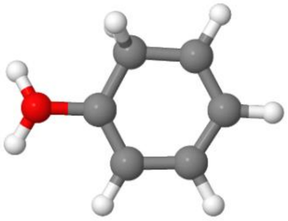

H-G

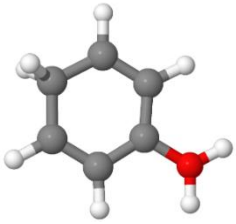

H-H

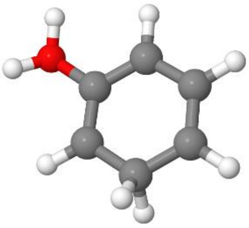

H-I

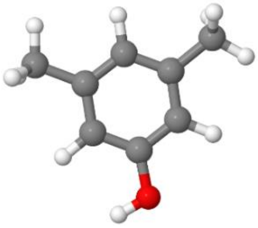

***m*Me-A**

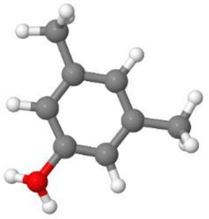

***m*Me-B**

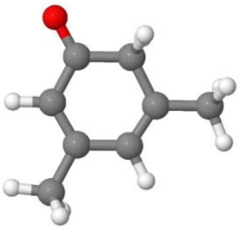

***m*Me-C**

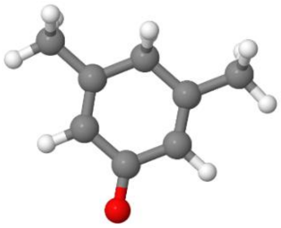

***m*Me-D**

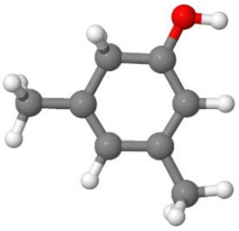

***m*Me-E**

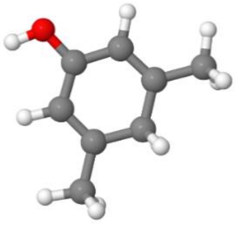

***m*Me-F**

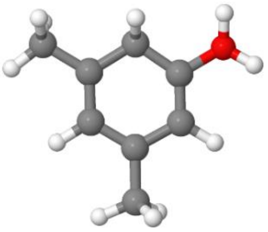

***m*Me-G**

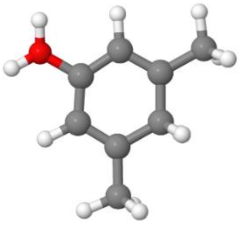

***m*Me-H**

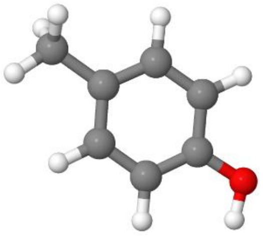

***p*Me-A**

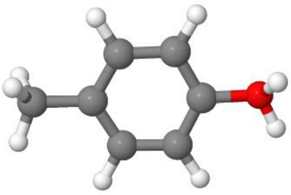

***p*Me-B**

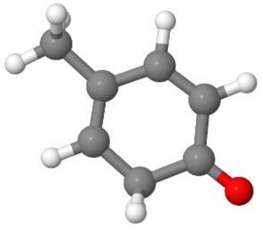

***p*Me-C**

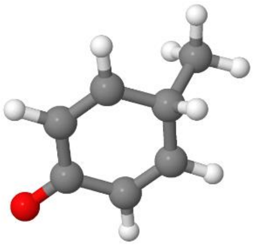

***p*Me-D**

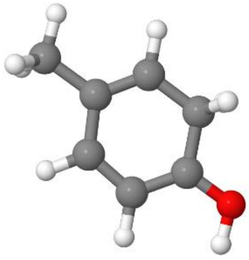

***p*Me-E**

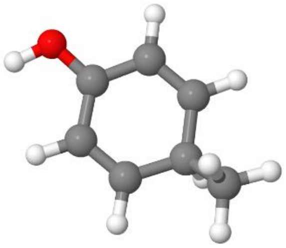

***p*Me-F**

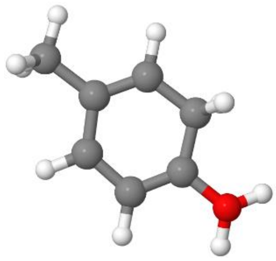

***p*Me-G**

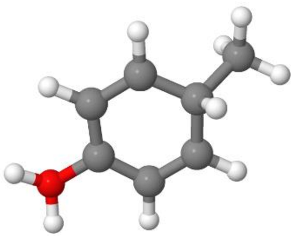

***p*Me-H**

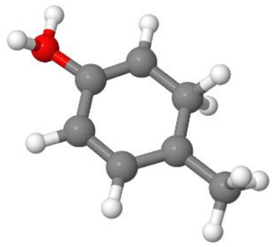

***p*Me-I**

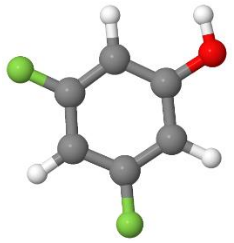

***m*F-A**

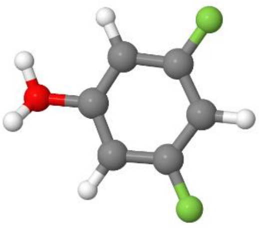

***m*F-B**

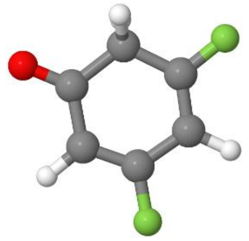

***m*F-C**

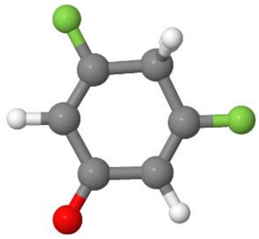

***m*F-D**

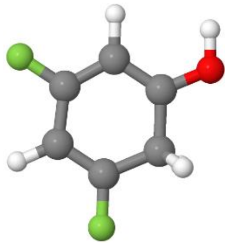

***m*F-E**

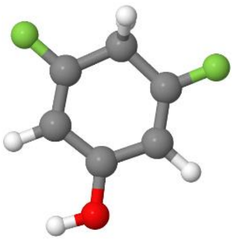

***m*F-F**

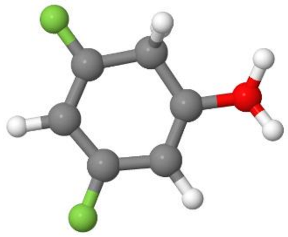

***m*F-G**

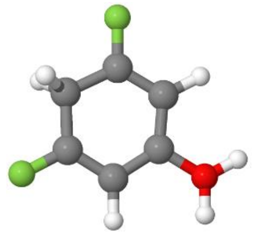

***m*F-H**

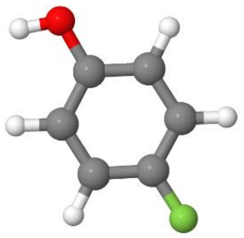

***p*F-A**

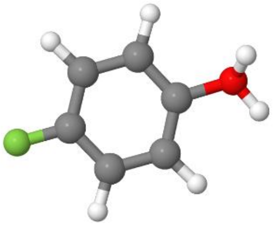

***p*F-B**

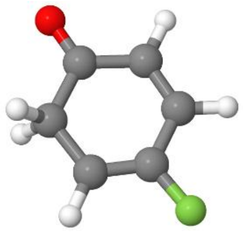

***p*F-C**

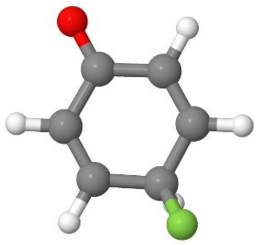

***p*F-D**

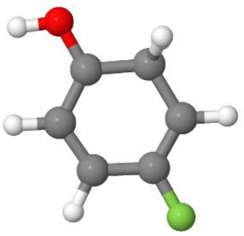

***p*F-E**

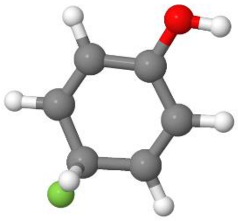

***p*F-F**

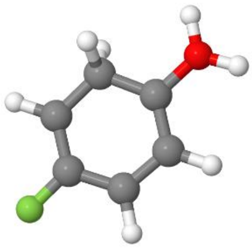

***p*F-G**

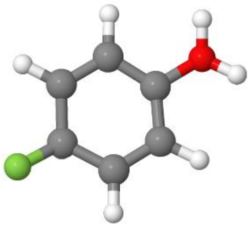

***p*F-H**

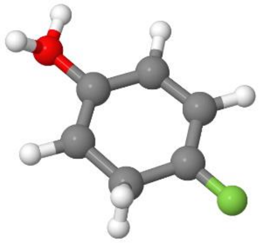

***p*F-I**

Figure S3. Calculated B3LYP/6-31G(2df,p) optimal geometries for phenol, ***m*Me-X**, ***p*Me-X**, ***m*OMe-X**, ***p*OMe-X**, ***m*OH-X** and ***p*OH-X** in all studied forms. The general geometries for F, Cl, Br and CN derivatives are analogical with parent phenol species and CF_3 derivatives are consistent with ***m*Me-X** and ***p*Me-X**. For cyano derivatives, the C(aromatic ring)–CN atoms are linearly arranged.

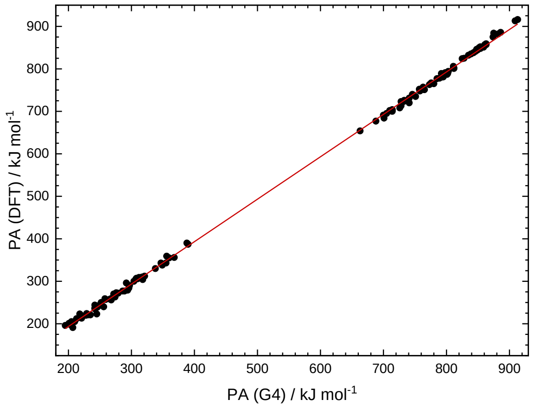

a)

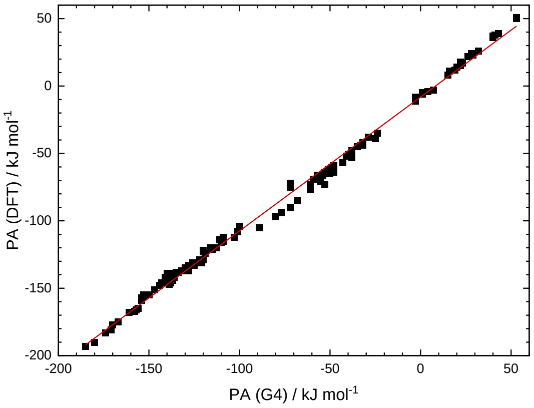

b)

Figure S4. Correlation between G4 and M06-2X/6-311++G(d,p) for proton affinities in the a) gas-phase with Intercept is –6.1 ± 1.2 and slope 0.9986 ± 0.0019 with R^2 = 0.999, b) water with Intercept is –8.19 ± 0.57 and slope 0.9935 ± 0.0058 with R^2 = 0.996.

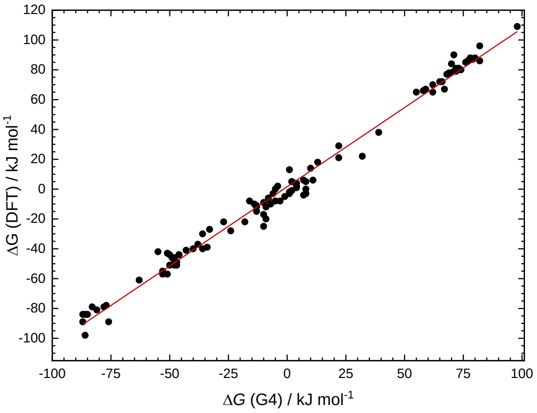

a)

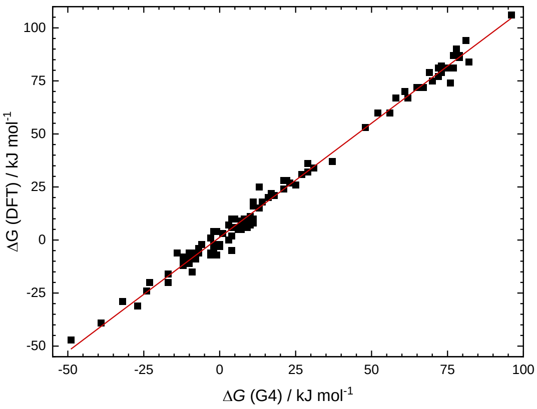

b)

Figure S5. Correlation between G4 and M06-2X/6-311++G(d,p) for Gibbs energies in the a) gas-phase with Intercept is 1.60 ± 0.55 and slope 1.061 ± 0.011 with R^2 = 0.989, b) water with Intercept is 1.28 ± 0.39 and slope 1.075 ± 0.010 with R^2 = 0.991.

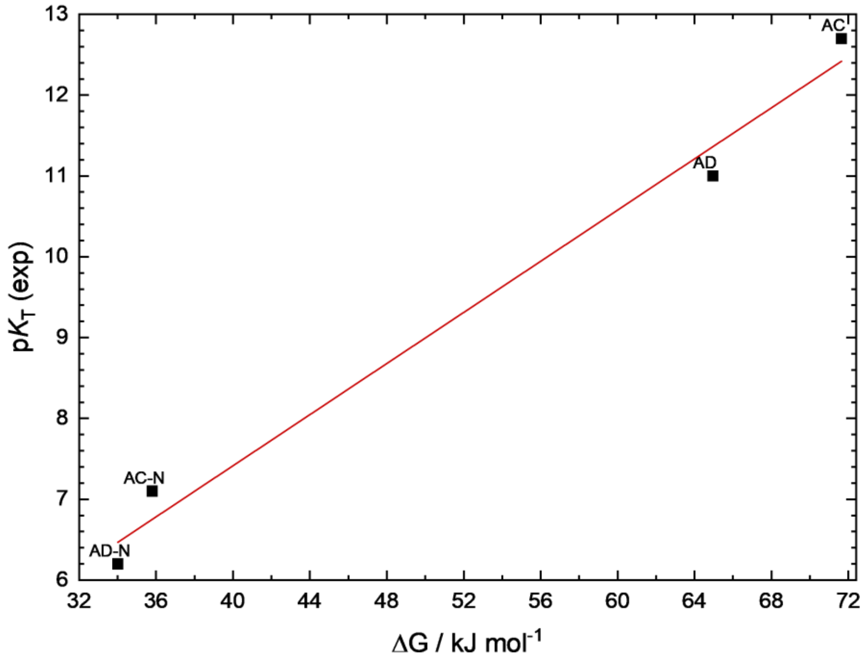

Figure S6. Correlation between available experimental pK_T values and calculation G4 Gibbs free energies of studied tautomerization reaction for naphthol (**AC-N**, **AD-N**) and phenol (**AC**, **AD**). Intercept is 1.09 ± 0.72 and slope 0.158 ± 0.013 with R^2 = 0.986.

Table S1. Calculated Gibbs free energies and experimental pK_T which was used in regression.

	**Δ*G* / kJ mol⁻¹**		**p*K*$_T$**	
**molecule/reaction**	**AC**	**AD**	**AC**	**AD**
1-naphthol	36	34	7.1[1]	6.2[1]
Phenol	72	65	12.7[2]	11.0[2]

Table S2. Calculated proton affinities (PA) of selected protonation reactions in gas-phase and water (in kJ mol^{-1}).

R	PA(AB)		PA(AE)		PA(AF)		PA(BG)		PA(BH)		PA(BI)		PA(CE)		PA(DF)		PA(EG)		PA(FH)	
	gas	water	gas	water	gas	water	gas	water	gas	water	gas	water	gas	water	gas	water	gas	water	gas	water
H	746	–35	798	–35	812	–24	297	–116	308	–109	307	–122	874	40	882	43	246	–115	242	–120
*m*Me	763	–32	849	–3	859	7	390	–72	388	–72			909	53	913	53	304	–101	292	–111
*m*Br	714	–53	794	–57	802	–48	368	–141	360	–139			863	16	863	15	289	–137	272	–144
*m*Cl	712	–51	793	–48	801	–38	355	–125	347	–123			861	23	861	22	274	–128	259	–136
*m*F	705	–51	789	–33	795	–25	318	–103	310	–109			855	32	852	29	235	–121	220	–135
mCF_3	688	–54	726	–80	741	–72	245	–180	256	–171			803	1	812	4	207	–154	204	–153
*m*CN	663	–59	701	–89	714	–80	243	–185	246	–174			785	–3	792	1	205	–154	195	–153
*p*Me	757	–32	811	–29	811	–32	321	–110	312	–119	356	–100	875	41	886	40	266	–113	258	–120
*p*F	733	–38	773	–48	751	–68	268	–136	231	–167	317	–120	848	28	835	16	228	–126	213	–137
*p*Cl	732	–40	775	–50	765	–61	286	–138	252	–161	338	–128	852	28	846	19	242	–128	218	–141
*p*Br	728	–41	776	–52	780	–55	296	–141	294	–157	349	–138	854	26	854	19	247	–130	242	–143
pCF_3	710	–43	758	–50	765	–53	269	–140	273	–150	279	–156	839	28	843	22	221	–132	218	–140
*p*CN	700	–49	741	–61	728	–77	251	–147	229	–170	276	–158	825	22	828	20	210	–134	201	–141

Table S3: Calculated M06-2X/6-311++G(d,p) proton affinities (PA) of selected protonation reactions in gas-phase and water (in kJ mol^{-1}).

R	PA(AB)		PA(AE)		PA(AF)		PA(BG)		PA(BH)		PA(BI)		PA(CE)		PA(DF)		PA(EG)		PA(FH)	
	gas	water	gas	water	gas	water	gas	water	gas	water	gas	water	gas	water	gas	water	gas	water	gas	water
**H**	**740**	–45	**791**	–45	**802**	–35	**290**	–120	**307**	–112	**305**	–129	**875**	37	**882**	39	**239**	–121	**244**	–123
**mMe**	**757**	–44	**844**	–11	**851**	–3	**387**	–75	**390**	–72			**913**	51	**916**	50	**300**	–108	**296**	–114
**mBr**	**704**	–64	**783**	–66	**789**	–64	**356**	–146	**355**	–147			**859**	11	**857**	8	**277**	–144	**270**	–148
**mCl**	**701**	–63	**781**	–60	**787**	–53	**343**	–133	**343**	–131			**857**	17	**855**	15	**263**	–136	**257**	–140
**mF**	**695**	–62	**778**	–44	**781**	–39	**304**	–112	**306**	–115			**851**	26	**848**	23	**221**	–131	**220**	–138
**mCF_3**	**677**	–66	**708**	–97	**720**	–90	**223**	–190	**240**	–181			**794**	–6	**801**	–4	**191**	–159	**197**	–156
**mCN**	**654**	–69	**684**	–105	**700**	–97	**236**	–193	**242**	–183			**777**	–8	**789**	–5	**205**	–157	**196**	–155
**pMe**	**752**	–42	**806**	–38	**802**	–43	**312**	–116	**309**	–124	**359**	–104	**884**	38	**886**	36	**258**	–120	**259**	–122
**pF**	**726**	–48	**763**	–59	**735**	–85	**256**	–142	**221**	–175	**310**	–129	**846**	24	**832**	11	**220**	–131	**212**	–139
**pCl**	**723**	–51	**765**	–62	**751**	–77	**277**	–143	**250**	–168	**330**	–137	**850**	24	**841**	12	**235**	–133	**223**	–142
**pBr**	**723**	–52	**767**	–63	**765**	–71	**284**	–146	**279**	–166	**338**	–146	**852**	22	**848**	12	**241**	–135	**237**	–146
**pCF_3**	**702**	–57	**748**	–65	**751**	–73	**260**	–145	**266**	–155	**272**	–165	**835**	23	**838**	16	**213**	–137	**217**	–139
**pCN**	**691**	–60	**731**	–73	**713**	–94	**245**	–151	**224**	–177	**273**	–167	**824**	18	**825**	14	**205**	–138	**201**	–143

Table S4. Calculated reaction M06-2X/6-311++G(d,p) Gibbs free energies ($\Delta_r G$) of studied tautomeric reactions in kJ mol^{-1}.

R	$\Delta_r G$(AC)		$\Delta_r G$(AD)		$\Delta_r G$(BE)		$\Delta_r G$(BF)		$\Delta_r G$(DC)		$\Delta_r G$(EF)		$\Delta_r G$(GH)		$\Delta_r G$(GI)		$\Delta_r G$(HI)	
	gas	water	gas	water	gas	water	gas	water	gas	water	gas	water	gas	water	gas	water	gas	water
**H**	79	77	78	72	–51	–3	–61	–11	1	5	–11	–8	–25	–9	–12	6	13	15
***p*Me**	77	75	86	82	–49	–4	–44	3	–10	–7	5	7	6	8	–40	–12	–46	–20
***p*F**	79	79	96	94	–37	8	–8	36	–17	–15	29	28	38	37	–51	–10	–89	–47
***p*Cl**	81	81	88	87	–41	7	–27	24	–8	–6	14	16	22	26	–57	–2	–79	–29
***p*Br**	80	81	81	81	–43	8	–42	18	–1	0	2	10	–2	20	–57	4	–55	–16
***p*CF_3**	84	74	90	81	–44	6	–44	10	–5	–7	0	4	–3	10	–10	21	–7	11
***p*CN**	87	86	109	106	–40	10	–22	32	–22	–20	18	22	21	27	–28	18	–49	–9
***m*Me**	67	60	65	53	–89	–31	–98	–39	2	7	–9	–8	5	1				
***m*Br**	72	79	67	70	–78	6	–84	1	6	9	–6	–6	–3	–2				
***m*Cl**	72	72	66	67	–79	–2	–84	–8	6	6	–6	–6	–4	–2				
***m*F**	70	67	65	60	–81	–20	–84	–24	5	8	–3	–4	0	2				
***m*CF_3**	85	87	81	82	–39	31	–51	25	4	5	–12	–6	–20	–9				
***m*CN**	88	84	86	90	–30	34	–46	28	1	–5	–15	–6	–8	–8				

References

(1) Gut, I. G.; Scheibler, L. C.; Wirz, J. Flash Photolytic Generation of Two Keto Tautomers of 1-Naphthol in Aqueous Solution: Kinetics and Equilibria of Enolization. *Photochem. Photobiol. Sci.* **2010**, *9* (7), 901–907.

(2) Capponi, M.; Gut, I. G.; Hellrung, B.; Persy, G.; Wirz, J. Ketonization Equilibria of Phenol in Aqueous Solution. *Can. J. Chem.* **1999**, *77* (5–6), 605–613.
